# Inflammation and α-Synuclein’s Prion-like Behavior in Parkinson's Disease—Is There a Link?

**DOI:** 10.1007/s12035-012-8267-8

**Published:** 2012-04-29

**Authors:** Carla M. Lema Tomé, Trevor Tyson, Nolwen L. Rey, Stefan Grathwohl, Markus Britschgi, Patrik Brundin

**Affiliations:** 1 Neuronal Survival Unit, Wallenberg Neuroscience Center, Lund University, BMC B11, 221 84 Lund, Sweden; 2 F. Hoffmann-La Roche Ltd, pRED, Pharma Research & Early Development, DTA CNS, Grenzacherstrasse 124, Basel, 4070 Switzerland; 3 Center for Neurodegenerative Science, Van Andel Research Institute, 333 Bostwick Avenue NE, Grand Rapids, MI 49503 USA

**Keywords:** α-Synuclein, Neuroinflammation, Prion-like, Parkinson's disease, Synucleinopathies, Dual-hit hypothesis, Proteinopathy

## Abstract

Parkinson’s disease patients exhibit progressive spreading of aggregated α-synuclein in the nervous system. This slow process follows a specific pattern in an inflamed tissue environment. Recent research suggests that prion-like mechanisms contribute to the propagation of α-synuclein pathology. Little is known about factors that might affect the prion-like behavior of misfolded α-synuclein. In this review, we suggest that neuroinflammation plays an important role. We discuss causes of inflammation in the olfactory bulb and gastrointestinal tract and how this may promote the initial misfolding and aggregation of α-synuclein, which might set in motion events that lead to Parkinson's disease neuropathology. We propose that neuroinflammation promotes the prion-like behavior of α-synuclein and that novel anti-inflammatory therapies targeting this mechanism could slow disease progression.

## Introduction

Age-related neurodegenerative disorders, including Parkinson’s disease (PD), are neuropathologically characterized by accumulation of misfolded proteins and the loss of neurons along with sustained neuroinflammation. Clinically, PD patients experience both motor (e.g., bradykinesia, resting tremor, rigidity, and postural instability) and non-motor symptoms (e.g., hyposmia, constipation, sleep disturbances, depression, and dementia). Age is considered the single greatest risk factor for developing PD with 4–5 % of people over 85 years developing the disease [1]. In PD, dopamine-producing neurons in the substantia nigra pars compacta (SNpc) die and there is a concomitant decrease of dopaminergic innervation in the striatum. Furthermore, alpha-synuclein (α-syn) misfolds and forms intraneuronal aggregates in several brain regions. α-Syn is a 140-amino acid protein encoded by a single gene located in chromosome 4 [2] and was first described to be present in the nucleus and presynaptic terminals of normal neurons [3]. In PD and the related synucleinopathies multiple system atrophy and dementia with Lewy bodies, misfolded and aggregated α-syn forms intra-cytoplasmic inclusions. In PD, they are located in neurons and are called Lewy bodies (LB) and Lewy neurites (LN) [4–6]. The idea that α-syn plays a central role in PD pathogenesis is supported by the link between three missense α-syn mutations, i.e., Ala53Thr [7], Ala30Pro [8], and Glu46Lys [9] and autosomal dominant early-onset PD. Furthermore, multiplications of the gene cause familial forms of neurodegenerative disease with parkinsonian features [10] and certain SNPs in the α-syn promoter are associated with increased risk for PD [11]. Recent studies suggest that prion-like cell-to-cell transfer of possibly misfolded α-syn contributes to the spreading of neuropathology from one brain region to the next [12–17].

Although there is no consensus on the causes of sporadic PD, neuroinflammation appears to play an important role (reviewed in [18]). Several questions still remain: how, if at all, is neuroinflammation linked to α-syn misfolding and aggregation? For example, does neuroinflammation trigger α-syn misfolding or does aggregated α-syn cause microglia activation and neuroinflammation? Furthermore, does neuroinflammation promote cell-to-cell transfer of α-syn and seeding and development of new α-syn aggregates? Alternatively, does the inflammatory process delay the prion-like spread of α-syn? In this review, we will discuss various aspects that drive pathological alterations of α-syn with a special focus on how neuroinflammation might affect the different steps.

## Involvement of Immune Pathways in the Brain of Patients with Parkinson’s Disease

Inflammation is a specialized reaction of the body to harmful agents or insults to tissue. Inflammatory processes in the nervous system are called "neuroinflammation" and they are characteristic to most neurodegenerative diseases, including PD. Classically, inflammation and neuroinflammation involve innate and adaptive immune responses that are regulated by secreted factors (e.g., cytokines, chemokines, complement proteins, acute phase proteins, reactive oxygen species (ROS), and arachidonate metabolites; reviewed in [19, 20]). Interestingly, genetic alterations in several immune function-related genes (e.g., DJ-1, leucine-rich repeat protein kinase-2 (LRRK2) and HLA-DR) can cause familial PD or increase the risk of developing PD (all studies and meta-analyses collected in www.pdgene.org). This points to a key role for immune pathways in the pathogenetic mechanisms of PD which are possibly triggered by aging, oxidative stress, or abnormal forms of α-syn (reviewed in [21]).

Microglia and astrocytes are resident immune cells of the brain. The expression of specific markers associated with these cells (typically Iba-1, CD11b, and MHC class II for microglia, GFAP for astrocytes) increases with severity of neuroinflammation. Positron emission tomography imaging studies employing microglia-specific markers point to an early involvement and cerebral propagation of neuroinflammation in PD [22, 23]. Histological analysis of the substantia nigra in PD patients shows activated microglia [24–26], a high density of astrocytes [27], and in some cases also infiltrating T lymphocytes [26, 28]. Furthermore, postmortem PD brains exhibit increased levels of tumor necrosis factor (TNF) [29] and interleukin (IL)-1 and IL-6 [30]. The cerebrospinal fluid (CSF) of PD patients differs from that collected from Alzheimer’s disease (AD) patients, other neurodegenerative disorders or healthy individuals [31]. The CSF levels of transforming growth factor β1 and 2 (TGFβ1 and TGFβ2) [32] and ratios between amyloid-β (Aβ) 42 with fractalkine/CX3CL1 [33], as well as levels of complement factors C3 and fH [34], are all altered in PD. Whether the clinical findings should be taken as evidence that neuroinflammation directly contributes to neurodegeneration in PD is not clear. The secreted factors and activated immune cells are not just potentially neurotoxic, but can also exert neuroprotective effects in neurodegenerative disorders (reviewed in [35, 36]).

In summary, signs of neuroinflammation and genetic links between PD and immune function point to a possible role of immune pathways in PD. Whether inflammation is beneficial or detrimental in this context remains unclear and we think it is pertinent to ask the question what role inflammation could specifically play in propagation of synucleinopathy in PD.

## Recent Updates About the Link Between α-Synuclein Aggregation and the Effect of Inflammation

It has been hypothesized that inflammation triggers, or at least contributes to, the development of α-syn pathology [12]. Indeed, in cellular assays, misfolded α-syn species can trigger activation of microglia [37–42], one of the hallmarks of the neuroinflammatory process. Although a specific receptor for α-syn binding to microglia is still unknown, these cells can take up extracellular α-syn [43, 44] that in turn triggers the release of soluble immune modulators. The vulnerability of dopaminergic neurons to inflammation has also been linked to α-syn, as genetic ablation of α-syn in animal models decreases the sensitivity of these cells to inflammatory challenges [45]. Factors released from activated microglia can further enhance oxidative stress, protein misfolding, and aggregation, creating a positive feedback loop promoting degeneration of dopaminergic cells [46, 47]. Together, it remains unclear whether α-syn aggregation is a cause or consequence of inflammation.

Approximately 90 % of α-syn found in LBs is constitutively phosphorylated at serine 87 (S87-P) and serine 129 (S129-P). By contrast, less than 5 % of α-syn is phosphorylated at serine 129 in healthy human brain [48–50]. Phosphorylation of α-syn in neuronal cell lines has been linked to detrimental activation of microglia in mixed cell culture systems [51], but whether phosphorylation of α-syn induces inflammation or vice versa is not fully understood. Furthermore, the precise roles of phosphorylation or other post-translational modifications of α-syn for neurotoxicity, formation of aggregates, and activation of microglia are not clear, with different studies reporting conflicting results [52–55].

Nitrated forms of α-syn have previously been suggested to be crucial to the pathogenesis of synucleinopathies [56] and nitrated α-syn can trigger inflammation and microglia activation [57, 58]. One study has shown that nitrated α-syn may be able to recruit peripheral leukocytes in cervical lymph nodes in a toxin-based (systemic injections of 1-methyl-4-phenyl-1,2,3,6-tetrahydropyridine, (MPTP)) mouse model of PD. The same study also shows that adoptive transfer of T cells from syngeneic mice immunized with nitrated α-syn worsens dopaminergic neuron loss after MPTP treatment of recipient mice [57]. This indicates that certain adaptive immune mechanisms could play a role in PD pathobiology.

A recent study suggests that α-syn may be modified by inflammation caused by expression of human wild-type or mutant P301L Tau [59]. Both forms of Tau cause microglial changes and increase IL-6 and TNF-α levels while also increasing the levels of endogenous α-syn and S129-P α-syn.

Inflammation and aggregation of α-syn are dynamically interlinked and this interaction probably plays a key role in the pathogenesis of PD. As discussed in this review, there is increasing evidence that α-syn aggregation may be initiated by a seeding mechanism that could spread throughout neurons in a prion-like fashion possibly involving other amyloidogenic proteins. For example, fragments of α-syn are commonly found in Aβ plaques in the brain of AD patients [60], and certain rare mutations leading to familial forms of AD have been associated with LB pathology as well [61, 62]. Aggregation of α-syn and Aβ can trigger neuroinflammation, e.g., it activates microglia (for reviews see [63, 64]). Oxidative stress produced by neuroinflammation is reported to be a cellular consequence of α-syn aggregation, and excessive levels of free ROS can trigger more inflammation and α-syn aggregation [65–67]. This vicious feed-forward loop of aggregation-induced neuroinflammation that in turn causes more aggregation may be a central mechanism in the pathogenesis of PD and other proteinopathies of the brain.

The NAD-dependent deacetylase, sirtuin 1 (SIRT1), has been recently identified as a possible regulator of α-syn aggregation. Sirtuins are known to have anti-aging effects in nematodes and it is suggested that this family of enzymes may also play an important role in the regulation of inflammation (reviewed in depth by Galli et al. [68]). Recently, it was demonstrated that overexpression of SIRT1 in A53T α-syn transgenic mice increases their lifespan and reduces α-syn aggregation, and conversely, knocking-out SIRT1 has the opposite effect [69]. The underlying mechanism has been suggested to be lack of SIRT1-regulated activation of molecular chaperons, which leads to aggregation of α-syn. Thus, sirtuins affect both α-syn aggregation and inflammation and could provide a molecular link between these two processes in PD.

The development and maintenance of dopaminergic neurons is regulated by the orphan receptor Nurr1 [70]. In addition to its role in neurons, loss of Nurr1 also disinhibits the NF-kB signaling cascade in astrocytes and microglia leading to increased secretion of neurotoxic mediators by these cells thereby promoting the loss of dopaminergic neurons in a mouse model of PD [71]. Rare mutations in Nurr1 have been associated with a late-onset familial form of PD, which presents with reduced expression of Nurr1 in somatic cells including peripheral immune cells [72]. This link between Nurr1, inflammation, and PD phenotype is intriguing, but it remains to be shown whether it is relevant to α-syn aggregation.

## Spread of Synucleinopathy and Progression of Parkinson’s Disease

The extent of α-syn pathology in the brain is believed to correlate to neurological disease stage [73]. Thus, just as the progression of symptoms tends to follow a predictable pattern, e.g., with certain non-motor symptoms preceding the onset of motor dysfunction, the progression of neuropathological changes are believed to follow a highly replicable pattern. Postulated by Braak and colleagues, this progression is divided into six stages in PD, starting first with the simultaneous appearance of α-syn aggregates in anterior olfactory structures and the vagal nerve. Thereafter, the pathology spreads rostrally, indicated by LNs and to some extent LBs. During stage 2, the pathology manifests itself in the caudal raphe nucleus, in the gigantocellular reticular nucleus, and in the coeruleus–subcoeruleus complex. It is not until stage 3 where it appears in the substantia nigra. At stage 4, lesions are detected in proencephalic regions and finally reach areas of neocortex and prefrontal cortex at the last stages [74]. This progressive pattern of appearance of α-syn aggregates in the brain suggests two possible routes for the spread of synucleinopathy into the brain following long and unmyelinated axonal projections, with a retrograde or anterograde progression [75]: first, the gastrointestinal route from the enteric nervous system (ENS) to the central nervous system (CNS) via dorsal motor neurons of the vagus (DMV) and the intermediolateral nucleus of the spinal cord [76–78]; and second, the olfactory route, from the olfactory bulb to the midbrain via other olfactory areas and the limbic system [79].

Several groups have debated the validity of the notion of progression of synucleinopathy and report a broad range of roughly 50–80 % of cases concurring with Braak’s staging [80–82] or concluding that it can be readily applied to most subjects [83]. In addition, other clinical studies on metabolic, structural, and functional data revealed that neuronal populations are differentially vulnerable to α-syn pathology and hence dysfunction is not in accordance to the proposed staging system [84]. Despite the controversy about the interpretation of neuropathology linked to clinical symptoms, these [80–82] and other studies [85–87] have led to the proposal of a unified staging system for all Lewy body disorders [88].

Interestingly, an estimated proportion of 90 % of PD patients exhibit olfactory deficits [89–91]. Several studies detected olfactory deficits early in the course of the disease, before the appearance of motor symptoms (for review, see [92]). Olfactory disturbances occur both in familial and sporadic forms of PD [90]. Histological studies in PD have also revealed a high degree of degeneration of olfactory cells, especially in the anterior olfactory nucleus (AON), which has been correlated to disease duration and LB load in the AON and olfactory bulb (OB) [90]. Thus, it has been proposed that PD might be primarily a disorder of olfaction [93] and that the olfactory pathways should be of high interest to study early PD stages.

Other histological studies in pre-symptomatic subjects, who go on to develop PD later in life, or PD patients with colonic bacterial infection, demonstrate pathological changes in the ENS that are associated with premotor symptoms involving the gastrointestinal (GI) tract and aggregation of α-syn in the ENS [94–97]. Indeed, the majority of PD patients display accumulation of pathology-related phosphorylated α-syn in enteric neurons [98]. In line with these findings, the DMV connecting the ENS to the CNS has long been known to be severely affected in PD, potentially influencing GI functions [74]. GI-related non-motor symptoms therefore might represent a prodromal stage of PD.

Together, these data suggest that olfactory and enteric regions might be the starting points of the disease that spreads then to the CNS. Based on this idea, Hawkes et al. proposed a dual-hit hypothesis: a neurotropic pathogen could attack the nervous system from two routes—anterogradely through olfactory tracts and retrogradely through the vagal nerve—and this pathogen could enter the brain through the olfactory and enteric epithelium [79, 99]. Apart from targeting neurons directly, it is quite possible that a pathogen triggers local inflammatory processes, which could initiate synucleinopathy in olfactory and enteric regions leading to a progression of pathology. In the following sections, we describe how inflammation in the olfactory system and in the GI tract might be triggers that start the process of α-syn aggregation.

### A Possible Role for Neuroinflammation in the Olfactory Bulb as a Trigger for PD

According to histological studies of PD patients' brains, α-syn abnormalities observed in anterior olfactory structures at Braak’s stage 1 are restricted to the AON and OB [74, 100]. More recent studies have demonstrated that after this step, the pathology spreads slowly into other olfactory regions like the piriform, periamygdaloid and entorhinal cortices, and the olfactory tubercle, without any progression in non-olfactory cortical areas [79].

Post-mortem studies investigating neuroinflammation in PD patients' brains have focused on midbrain structures. More extensive studies reported activated microglia also in the hippocampus and the temporal and entorhinal cortices [24]. Despite the involvement of olfactory regions in PD and the importance of neuroinflammation in PD, only one study has focused on the OB and demonstrated microgliosis in the OB of PD patients [101]. Due to its proximity to the olfactory epithelium and connections with the periphery, the olfactory system is very sensitive to exogenous pathogens, which might trigger local inflammation.

#### Sensibility of the Olfactory Epithelium and Olfactory Bulb to Inflammation

The olfactory epithelium is the first point of contact with the external environment. Mucocillaries of the olfactory epithelium act as a partial barrier against exogenous particles in the air [102, 103], but do not provide complete protection (for review see [104]). In humans, the thickness of the olfactory epithelium declines during aging [105] and thereby it might also become more permeable to particles and pathogens, increasing the risk of brain infections.

The organization of the human and rodent olfactory systems is similar [106] despite olfactory functions being less well developed in humans. In both species, the olfactory epithelium neurons, in contact with the external environment, project their axons into the glomerular layer of the OB, where they contact mitral cells, the relay cells of olfactory information. In rodents, these two types of cells have been shown to constitute a route for pathogen entry into the brain [104].

Further, in rodents, the OB contains a very dense population of microglial cells compared to other structures [107, 108], especially in the glomerular layer. A possible role of microglia in the OB is to phagocytose cellular debris [109] resulting from the high turnover of olfactory receptor neurons in the olfactory epithelium. Moreover, the microglia may also phagocytose OB interneurons [110] which are replaced throughout life in rodents, as well as possibly in humans, by adult neurogenesis [111–113]. Finally, OB microglia might also function as a sensor and barrier to exogenous particles and pathogens and could be involved in modulation and potentiation of neuroinflammation and associated disease [114, 115].

Among exogenous particles that can enter the OB, toxins (for review, see [116]), viruses, and particles (ultra-thin particles <100 nm, nanoparticles and microparticles) [99, 115, 117–121] have been widely studied. Studies in animal models have demonstrated that pathogens can be either blocked in the glomerular layer of the OB or spread though the axons of mitral cells to higher olfactory structures, such as the olfactory tubercle, amygdala, and olfactory cortex [114, 115].

#### Viruses Causing Inflammation in Olfactory Structures

The idea of a viral origin of PD was initially based on the observation that people born during the influenza pandemic 1918–1919 exhibit increased risk of developing parkinsonism, or so called post-encephalitic PD [122]. Viral infections during childhood have also been generally associated with higher likelihood of developing PD [123]. Moreover, diseases like asthma and seasonal and allergic rhinitis might be associated with PD [124], suggesting that chronic rhinitis, i.e., rhinitis-related inflammation, might promote the entry of neurotropic pathogens via the olfactory route [79]. Interestingly, influenza A virus has been detected in the SNpc of PD brains [125], and the interferon-induced protein MxA, which plays a role in defense against influenza virus, is associated with LBs [126]*.*

The infection by neurotropic viruses through the olfactory route has been widely studied in experimental animals. Rodent studies demonstrate that various types of viruses entering via the olfactory epithelium can infect different cell types in the OB, leading to dysfunction in olfactory circuits, and to the activation of glial cells located in the outer layers of the OB [114, 117, 119, 127–131]. Depending on the viral dose [115], an immune reaction involving type I interferons (e.g., IFN-α and IFN-β) can sometimes block the propagation of the virus from the OB to other brain regions [132], but the immune reaction can also induce widespread inflammation. The effects of influenza virus infection on olfactory regions have been well described in rodents [117, 128, 133]. Shortly after infusion of a mouse-adapted human influenza virus in the nasal cavity of mice, the viral antigen is found in the OB (and more precisely in glial cells), but not in other brain regions. Production of cytokines, such as IL-1β and TNF-α, increased considerably at the onset of disease. The neuronal release of TNF-α and IL-1β was also increased in other brain regions, including those directly connected with the OB (piriform cortex, olfactory tubercle, central amygdala) and others, such as the hypothalamus. Hence, local infection in the OB can induce inflammatory signals in distant brain regions and can trigger global neuroinflammation.

As mentioned above, activated microglia can phagocytose pathogens and unwanted cells [134] acquire an antigen-presenting cell phenotype and contribute to the recruitment of T cells [135]. ROS and cytokines produced by microglia disrupt the blood brain barrier (BBB) [136, 137], and the effect of these inflammatory mediators is more pronounced in the olfactory epithelium and OB where the BBB already is more permeable than the BBB of other structures [138–141]. Recently, the effect of TNF-α on BBB permeability was shown to favor CNS infection by the West Nile virus in mice [142]. Thus, neuroinflammation in the OB might increase BBB permeability, facilitating the penetration of exogenous pathogens and triggering microgliosis and leukocyte invasion into the OB, sparking off a vicious circle of inflammation. Possibly, a similar mechanism plays a role in humans, after a neurotropic virus infection entering through the nose.

Recently, Jang and colleagues [143] studied the consequences of H5N1 viral infection in C57Bl/6 mice by intranasal inoculation. The H5N1 avian influenza virus spread from the periphery to the CNS (via cranial nerves) and was found in neurons and astrocytes in the solitary nucleus, locus coeruleus, and in periglomerular and mitral cells of the OB. Twenty-one days post inoculation, the virus was no longer found in the brain, but the immune response persisted at least 2 months. Importantly, this study was the first to demonstrate the effect of viral infection on α-syn aggregation. An increase in cellular and secreted phosphorylated α-syn (pSer129) was observed in the OB, locus coeruleus, hippocampus, and brainstem. Moreover, aggregated α-syn was found in the hippocampus, cortex, and brainstem along with a loss of dopaminergic neurons in SNpc [143]. Taken together, these data point to the possibility that viral infection, maybe through inflammatory processes, can increase α-syn phosphorylation and induce/increase aggregation of α-syn in the brain.

#### Micro- and Nanoparticles Trigger Oxidative Stress and Inflammation in the OB

Certain metal particles might also be involved in PD, for example, LBs in the brain of PD patients have a high content of aluminum [144], or an enhanced risk to develop PD has been reported for subjects on a diet containing high quantities of iron [145]. Several studies have demonstrated that various types of particles (gold, ^13^C, manganese oxide, titanium dioxide, ferric oxide) can be inhaled, deposited on olfactory epithelium, tracheobronchial epithelium, and in lung alveoli in humans and mice (for review, see [121, 146]). Some particles then reach the CNS through olfactory epithelium and OB [120, 147–151] and may trigger inflammatory processes in the olfactory circuits. For example, in mice, an intranasal administration of Fe_2_O_3_ induces microglial proliferation, activation, and recruitment in the OB. In vitro, exposure to Fe_2_O_3_ leads to the release of ROS and NO by a microglia cell line that at the same time increases their proliferation [151]. Thus, chronic exposure to air particles, particularly in a polluted environment, could induce a chronic state of inflammation in olfactory and brain structures (for an extensive review on the involvement of viruses, particles, and pesticides in PD, see [152]).

#### Inflammation Process in the Olfactory Bulb: Local Consequences

Peripheral injection of lipopolysaccharide (LPS) is a widely used, but not very well understood, method to induce distant immune reactions in the rodent brain. Mori et al. and Ota et al. focused on olfactory regions and showed in mice a significant loss of cells in the OB and an increase in TNF-α and IκBα expression after peripheral LPS injection [153, 154]. Additionally, LPS administration increased expression of TNF receptors 1 and 2, and caspase 8 gene [154], as well as enhanced Fas expression [153]. Two other studies showed that TNF-α itself has deleterious effects on olfactory cells, causing dysfunction of olfactory neurons [155] and inhibiting regeneration of olfactory mucosa [156]. Moreover, when local inflammation in the OB after nasal instillation of a mycotoxin is coupled to a peripheral LPS injection, the expression of immune-stimulating genes is dramatically increased in the olfactory mucosa and the OB. This suggests that the effects of peripheral LPS could magnify apoptosis and inflammation in the olfactory epithelium and OB [118].

**Fig. 1:**
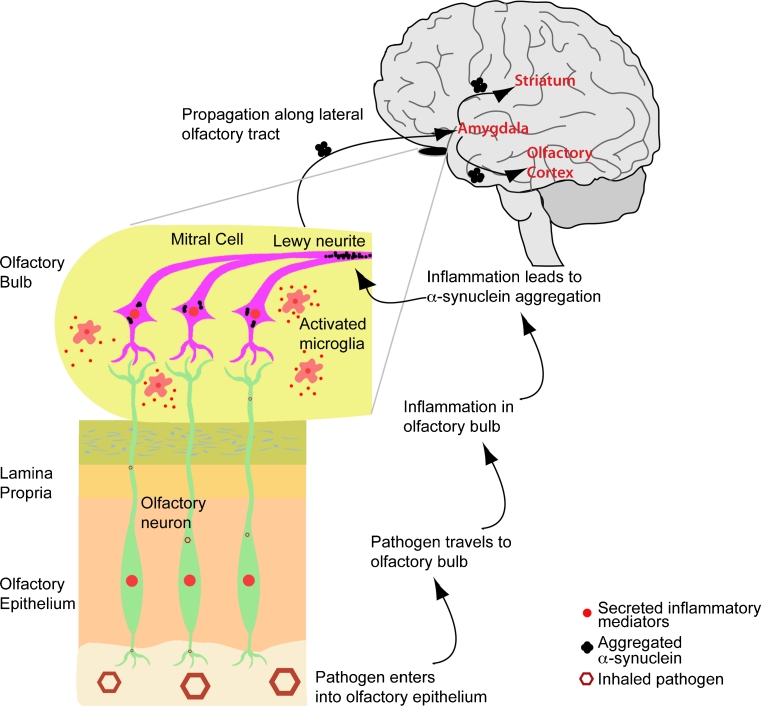
Possible initiation mechanism and spreading of synucleinopathy from the olfactory bulb to the brain. Pathogens or particles entering the olfactory epithelium spread to the olfactory bulb (OB) through axons of olfactory neurons. The pathogens induce an inflammatory response and oxidative stress in the OB and damage that accumulates overtime and induces α-syn misfolding and aggregation. α-Syn then transfers to interconnected regions via prion-like mechanisms and reaches the midbrain. General inflammation could promote α-syn accumulation and spread

In summary, local inflammatory processes can lead to neuronal death and olfactory neuron dysfunction, which may translate into early olfactory impairments occurring in early PD. Thus, exposure to viruses and micro/nanoparticles during our lifetime may initiate chronic inflammation through the olfactory network, induce aggregation of α-syn, and cause damage and dysfunctions that accumulate over time. This may enhance early synucleinopathy in OB and contribute to PD initiation and progression (see Fig. 1).

### Neuroinflammation May Promote Progression of Synucleinopathy from the Gastrointestinal Tract to the CNS

**Fig. 2:**
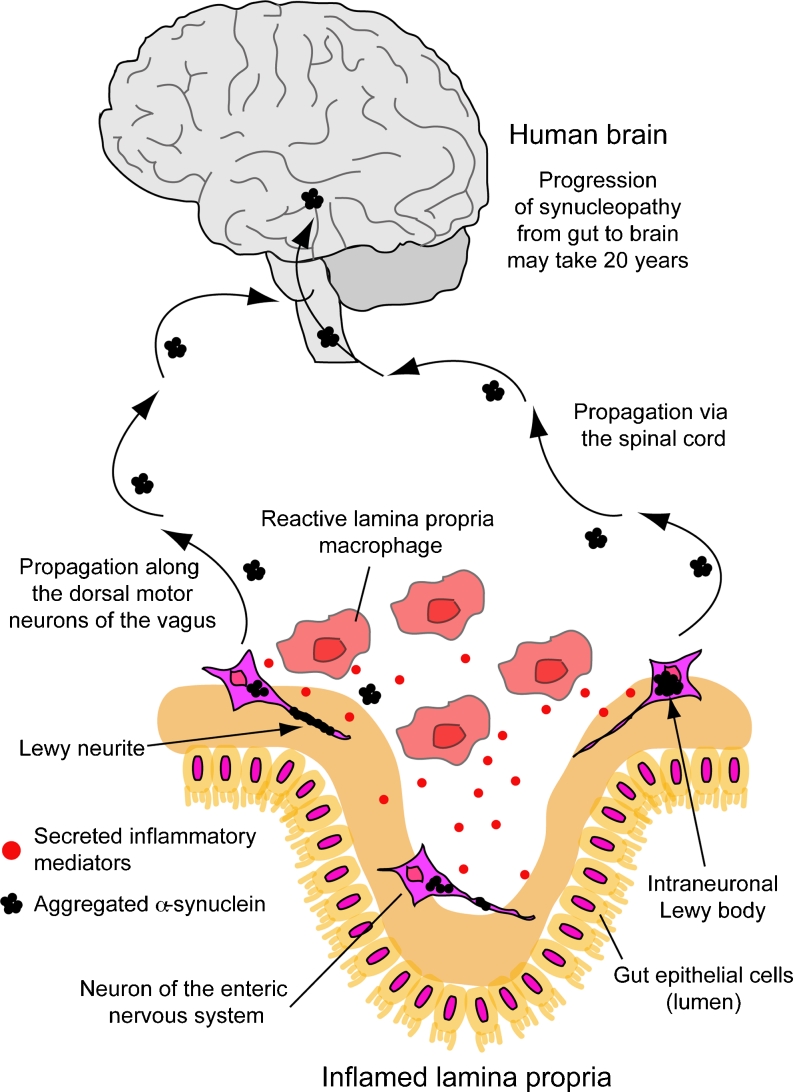
Possible spreading of synucleinopathy from the enteric nervous system to the brain. Macrophages in the lamina propria become reactive upon natural (e.g., bacteria, viruses, etc.) or induced (toxins) immune challenges. These cells react with secretion of inflammatory mediators (cytokines, chemokines, ROS, etc.), which can harm the surrounding tissue and may induce accumulation of α-syn in enteric nerves. This could alter gut activity, which early on may be observable by abnormal intestinal motility and constipation. Aggregated α-syn may be released by damaged nerve cells, which may further activate local macrophages. Cell-to-cell transmission could further contribute to the progression of synucleinopathy, which would eventually propagate from the enteric nervous system into the SNpc via nuclei in vagus and spinal cord

A growing body of evidence suggests that early on in PD, the peripheral nervous system and organs other than the brain are affected by neuropathological and neuroinflammatory events. Pathomechanisms in the ENS have raised interest in PD research due to distinct non-motor symptoms, such as dysphagia, constipation, and gastroesophageal reflux, experienced by PD patients [95, 157–161] and, interestingly, by certain elderly healthy individuals with no history of PD [96, 162–164]. Careful histological studies by Braak and colleagues support the hypothesis that accumulation of α-syn in the ENS could occur 20 years before the onset of degenerative changes in the CNS and associated motor symptoms [76, 94, 158, 164, 165]. The spread from the ENS to the CNS was proposed to occur via the DMV and the intermediolateral nucleus of the spinal cord [76, 166, 167]. Very little is known about the mechanisms that may promote this propagation. For example, a prion-like cell-to-cell progression along nerve bundles of the vagus nerve and spinal cord has been suggested [168, 169], which, similar to prion disorder, could involve immune pathways as well [170] (Fig. 2). This finds some clinical support in a study of patients with early stage diagnosed PD, where α-syn staining in the ENS correlated with compromised intestinal barrier integrity. Possible drivers of the observed pathology may have been bacterial and environmental toxins, resulting in increased oxidative stress most likely produced by macrophages in the luminal wall [97]. Yet in another study, patients with prolonged inflammation due to chronic inflammatory bowel disease did not display colonic synucleinopathy [171]. These findings in human patients indicate that acute local immune effects and certain forms of chronic intragastric inflammation may contribute differently to synucleinopathy in the GI.

In order to model some of the putative mechanisms in vivo and to validate clinical observations, appropriate rodent models reflecting the symptoms found in possible early stage diagnosed PD were studied [172]. Some transgenic mice expressing mutant human α-syn encoded on artificial chromosomes reveal early aggregates of α-syn in the ENS and display motor deficits prior to detectable pathology in the CNS [172, 173]. In rats, intragastric injection of a selective proteasome inhibitor induced α-syn aggregation in DMV [174]. In other studies, chronic intragastric administration of the mitochondrial toxin rotenone to rats was reported to induce a progressive accumulation of endogenous α-syn starting in the ENS and reaching the brain along the vagal and spinal cord nerve connections to the substantia nigra [175]. Neuropathological changes in the toxicity-induced PD rat models were accompanied by local neuroinflammation. It can be assumed that the resulting tissue destruction and rotenone directly triggered a local immune activation and together this may have propagated the pathology from the GI system to the brain.

In support of a direct link between PD and gut immune status, it was found that the gene LRRK2, which is the major genetic cause for familial PD [176–180], is also located in a risk region for Crohn’s disease [181], an autoimmune-mediated chronic inflammatory bowel disease. In the brain, neuronal overexpression of LRRK2 accelerated the development of neuropathology in A53T α-syn transgenic mice, whereas its ablation suppresses neuronal aggregation and cytotoxicity of α-syn [182]. In the peripheral immune system, LRRK2 is upregulated in macrophages under inflammatory conditions which promotes the production of ROS, phagocytosis, and killing of bacteria [183]. The link between genetic risk for PD and GI immune mechanisms for LRRK2 is intriguing and warrants further studies to identify possible associations with progression of α-syn aggregation from the ENS to the brain.

Before doing so, a better understanding of the GI immune system is required. The intestines, and in particular the subepithelial lamina propria, contain the largest pool of tissue macrophages in humans. Thereby, the intestines share physiological similarities with the immune system of the olfactory epithelium. Macrophages in both locations demonstrate a two-edged sword: on one hand, they maintain tissue homeostasis by active phagocytosis of invading pathogens (e.g., bacteria, viruses) in the absence of excessive activation of other immune pathways [108, 166, 184]; on the other hand, they bear the potential to release an arsenal of detrimental cytokines, chemokines, and ROS [179, 180]. Thus, depending on the circumstances, GI macrophages have the potential to harm the surrounding tissue and may play a role in promoting the accumulation of α-syn in enteric nerves, leading to altered gut motility and constipation and, in later stages, to propagation of the pathology to the brain. If this indeed constitutes an etiological trigger in PD, then it could be relevant for developing earlier treatments and diagnosis of the disease.

## Mechanisms of Cell-to-Cell Transfer

**Fig. 3:**
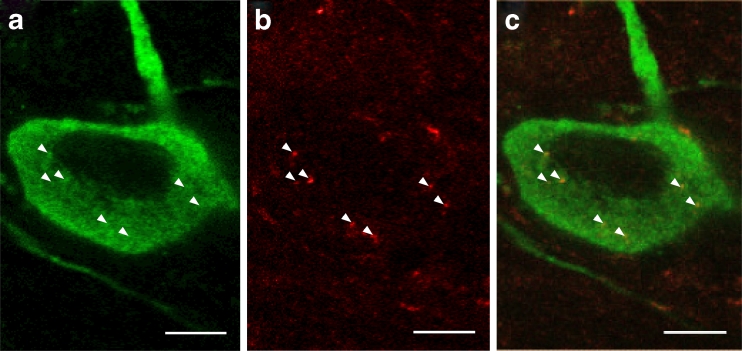
α-Syn propagates from mouse brain to a graft of dopaminergic neurons. Confocal planes of a tyrosine hydroxylase-positive mouse neuron (*green*, **a**) transplanted in the striatum of a mouse overexpressing human α-syn. The *arrowheads* indicate the localization inside the grafted mouse cell of several human α-syn (*red*, **b**) dots, which have been transferred from the host brain. **c** An overlay of **a** and **b**. *Scale bar* 5 μm. Figure courtesy of Dr. Elodie Angot

Post-mortem studies on PD patients who had received intrastriatal grafts of embryonic dopaminergic neurons over 10 years prior to death showed that some of the grafted neurons exhibit LBs and LNs cells [14, 16, 17, 185]. These findings stimulated the hypothesis that misfolded α-syn transferred directly from host brain cells to the grafted neurons. In support of this hypothesis, experiments performed in vitro [186, 187] and in vivo [13, 15, 188] have demonstrated that α-syn indeed can transfer between cells (Fig. 3) in a prion-like fashion. This is of direct relevance for how α-syn pathology might spread throughout the brain during the natural course of PD [12, 75, 168, 189]. For a comprehensive account of what is known about the underlying cellular and molecular mechanisms of cell-to-cell transfer of α-syn, we refer readers to other recent review articles on the topic [190–192] as it is beyond the scope of the current article. In brief, endocytosis [13, 193, 194] is strongly implicated as a α-syn uptake mechanism, and exosomes have been suggested to play a role when α-syn exits from cells. It has also been speculated that direct cell-to-cell transmission of α-syn occurs via tunneling nanotubes (tubes with a diameter <200 nm and containing F-actin) because a prior study demonstrated transfer of prion-protein via such structures [195]. Currently, the evidence linking α-syn misfolding or aggregation with cell-to-cell transfer is largely circumstantial. More studies are required to determine whether an increase of aggregated α-syn in a model system leads directly to an increase of the transfer rate of the protein. These studies are currently ongoing within our and other laboratories and should provide the evidence required to validate this hypothesis. Furthermore, the effects of neuroinflammation on the aforementioned cellular mechanisms are not known and inflammation might affect the rate of cell-to-cell α-syn transfer in either direction. For example, it is conceivable that some aspects of inflammation mitigates interneuronal α-syn transfer because activated microglia may phagocytose extracellular α-syn [44], thereby reducing the likelihood of neuron-to-neuron transfer of pathological α-syn. This hypothesis, however, remains to be tested. On the other hand, it has become clear that exposure of glial cells to α-syn or uptake of α-syn by astrocytes can turn their phenotype into a potentially more detrimental one for neurons [196, 197].

## Conclusions and Proposed Hypothesis

Although α-syn aggregation appears to be central to the development and progression of PD pathology, inflammation also plays an important role in this disease. Immune activation in the GI tract or in the olfactory system via chemical or virus exposure might trigger α-syn misfolding, subsequent aggregation, and propagation to the brain. It is also possible that neuroinflammation promotes the prion-like transfer of α-syn by increasing its release, increasing its uptake, or both. However, further studies are needed to support this hypothesis. If correct, it will open up new avenues for early detection and therapeutic intervention. For example, one could speculate that it will be possible to develop therapies which slow down the progression of PD by reducing the underlying inflammation and mitigating its effects on cell-to-cell α-syn transfer. In addition, it is conceivable that it would be possible to adopt a preventive strategy and lower the risk of developing PD by treating the triggers of inflammation in the olfactory or gastrointestinal system.
